# Whole mitochondrial genome analysis in highland Tibetans: further matrilineal genetic structure exploration

**DOI:** 10.3389/fgene.2023.1221388

**Published:** 2023-11-14

**Authors:** Xin Li, Xianpeng Zhang, Ting Yu, Liping Ye, Ting Huang, Ying Chen, Shuhan Liu, Youfeng Wen

**Affiliations:** ^1^ Institute of Biological Anthropology, Jinzhou Medical University, Jinzhou, China; ^2^ Department of Pathophysiology, Jinzhou Medical University, Jinzhou, China

**Keywords:** Tibetan, mtDNA, haplogroup, maternal, genetic structure

## Abstract

**Introduction:** The Qinghai–Tibet Plateau is one of the last terrestrial environments conquered by modern humans. Tibetans are among the few high-altitude settlers in the world, and understanding the genetic profile of Tibetans plays a pivotal role in studies of anthropology, genetics, and archaeology.

**Methods:** In this study, we investigated the maternal genetic landscape of Tibetans based on the whole mitochondrial genome collected from 145 unrelated native Lhasa Tibetans. Molecular diversity indices, haplotype diversity (HD), Tajima’s D and Fu’s Fs were calculated and the Bayesian Skyline Plot was obtained to determining the genetic profile and population fluctuation of Lhasa Tibetans. To further explore the genetic structure of Lhasa Tibetans, we collected 107 East Asian reference populations to perform principal component analysis (PCA), multidimensional scaling (MDS), calculated Fst values and constructed phylogenetic tree.

**Results:** The maternal genetic landscape of Tibetans showed obvious East Asian characteristics, M9a (28.28%), R (11.03%), F1 (12.41%), D4 (9.66%), N (6.21%), and M62 (4.14%) were the dominant haplogroups. The results of PCA, MDS, Fst and phylogenetic tree were consistent: Lhasa Tibetans clustered with other highland Tibeto-Burman speakers, there was obvious genetic homogeneity of Tibetans in Xizang, and genetic similarity between Tibetans and northern Han people and geographically adjacent populations was found. In addition, specific maternal lineages of Tibetans also be determined in this study.

**Discussion:** In general, this study further shed light on long-time matrilineal continuity on the Tibetan Plateau and the genetic connection between Tibetans and millet famers in the Yellow River Basin, and further revealed that multiple waves of population interaction and admixture during different historical periods between lowland and highland populations shaped the maternal genetic profile of Tibetans.

## 1 Introduction

East Asia is one of the most densely populated regions in the world, with rich genetic, cultural, and linguistic diversity. Paleogenomic studies have revealed a complex demographic history of migration, interaction, and admixture in East Asia (Ning et al., 2020; Yang et al., 2020), and there is long-time genetic continuity between ancient and modern East Asian populations, so investigation of the genetic structure of modern East Asian populations plays a pivotal role in comprehensively understanding population migration patterns and genetic relationships. The Sino-Tibetan language family is mainly distributed in East Asia, and the number of Sino-Tibetan language speakers is the largest in East Asia. The origin and dispersal of the Sino-Tibetan language are controversial. Recently, multidisciplinary studies have supported the northern-origin hypothesis of the Sino-Tibetan language family; the diffusion of the Sino-Tibetan language was concomitant with the diffusion of agriculture, and Neolithic millet farmers in the Yellow River Basin (YRB) may be the ancestral group of modern Sino-Tibetan speakers (Ning et al., 2020; Zhang et al., 2020; Wang C. C. et al., 2021). According to ancient DNA results, the modern Han people can be modelled as a mixture of YRB farmers and different indigenous populations. For example, excepting YRB-related ancestry, the northern Han people have more ancient Northeast Asian-related ancestry (Zhou et al., 2022), and the southern Han people show a greater genetic contribution of Yangtze River Basin ancestors (Huang et al., 2022). Studies of uniparental markers and analysis of genome-wide data have indicated that the genetic structure of Han people is characterized by a north–south genetic cline (Qin et al., 2014; Chiang et al., 2018). However, the fine-scale genetic structure of other Sino-Tibetan speakers is still unclear.

Tibetans belong to the Tibeto-Burman language group in the Sino-Tibetan language family. Tibetans are among the few high-altitude settlers in the world, and the adaption of high altitude in Tibetans can be attributed to archaic introgression and natural selection (Simonson et al., 2010; Zhang X. et al., 2021; Zhang P. et al., 2022). The Tibetan Plateau (TP) is one of the last populated areas of modern humans, and humans have inhabited the Qinghai–Tibet Plateau since 40.0–30.0 thousand years ago (kya) (Zhang et al., 2018), but evidence for permanent settlement dates to only 3,600 years before the present (BP), with the advent of agriculture (Chen et al., 2015). Previous studies have described the chronological profile of the TP’s colonization and have shown that the first migration occurred during the Upper Paleolithic rather than the Neolithic (Qi et al., 2013; Lu et al., 2016). The origin of Tibetans is more complicated and ancient; Paleolithic and Neolithic ancestries were found in the gene pool of Tibetans (Lu et al., 2016). Modern Tibetans can be modelled as a mixture of Upper Paleolithic TP inhabitants and later YRB farmers (Ning et al., 2020). Modern Tibetans exhibit northern East Asian affinity in genetic structure and physical characteristics, and genetic and physical similarities can be found between Han people and Tibetans (He et al., 2021; Li et al., 2022), revealing YRB millet farmers as the potential common ancestor group. There exists high genetic diversity in modern Tibetans, and excepting YRB-related ancestry, there is additional genetic influx from Siberia, western Eurasia, Central Asia, and South Asia (Lu et al., 2016; He et al., 2021; Liu et al., 2021), which sheds light on multiple waves of population migration toward the TP. A previous study also showed the genetic sub-structure of Tibetans, which is consistent with their cultural background and geographic distribution (He et al., 2021). In general, previous studies have indicated a complex dynamic population migration and admixture history, and investigating the genetic makeup of Tibetans plays a pivotal role in understanding demographic history and population structure in East Asia.

Mitochondria are indispensable organelles for energy production in eukaryotic cells (Vercellino and Sazanov, 2021). The mitochondrial genome has a higher mutation rate than the nuclear genome, and mtDNA mutations are an important cause of many diseases (Mustafa et al., 2020). A previous study revealed that the number of possible pathogenic mutations carried by highlanders, such as Tibetans, was significantly higher than that carried by lowland peoples (Kang et al., 2016). Meanwhile, mtDNA contains maternal genetic information and provides a distinctive perspective on the matrilineal aspects of population history. Previous studies have shown that the maternal genetic profile of Tibetans exhibits northern East Asian characteristics, and there is a similarity in maternal makeup between Tibetans and Han people (Wang et al., 2020). Haplogroup M9 is the main haplogroup in Tibetans (Kang et al., 2016), the frequency of M9 in the Tibetan gene pool is obviously higher than that in other East Asians, such as Han people (Gu et al., 2012), and the sub-haplogroup M9a1a1c1b1a is common in present-day Tibetans, which originated in northern China between 10.0 and 6.0 kya (Li, 2019). Mitogenome evidence indicates that Tibetan ancestors settled on the TP in the Late Paleolithic (Zhao et al., 2009; Qin et al., 2010), and the East Asian-related ancestry of modern Tibetans may have come from Neolithic YRB millet farmers (Li, 2019).

Diverse genetic ancestral sources and a complex demographic history of Tibetans have been revealed by previous studies (Bhandari et al., 2015; He et al., 2021; Zhang et al., 2023). Although many genetic studies on Tibetans have been published recently, their population genetic structure, spatiotemporal differentiation, and demographic history are still ambiguous and remain to be studied in depth. Genome-wide data, Y chromosomes, and mtDNA provide different perspectives for exploring population history, and a greater number of SNPs, larger sample sizes, and more sampling sites are key to further investigating fine-scale genetic structure and detailed demographic history. Therefore, we recruited 145 unrelated native Tibetan volunteers in Lhasa, which is the core region of the TP, and obtained their whole mitochondrial genome to explore genetic structure and population history for matrilineal insights.

## 2 Materials and methods

### 2.1 Study population

This study, which included peripheral blood samples from 145 unrelated healthy Tibetans, was carried out via health examination in August 2016 in Lhasa (altitude of 3,600 m), China. Our study was reviewed and approved by the Medical Ethics Committee of Jinzhou Medical University (JZMULL2016010) and followed the recommendations provided by the Helsinki Declaration. All Tibetan participants were sampled after obtaining verbal and written informed consent. To keep the representativeness of the samples, the Tibetan participants in this study were unrelated indigenous people who had lived in the sampling place for at least three generations, and they declared that no migration events had occurred in their family history.

### 2.2 DNA extraction, PCR amplification, and subsequent genotyping of mtDNA

DNA extraction was performed using the Eltbio^®^ Mag Blood DNA Small Extraction Kit (LD764), which is based on magnetic bead-based extraction, following the manufacturer’s instructions. The quantity of gDNA was measured using a NanoDrop ND-1000 instrument (NanoDrop Technologies, Wilmington, DE, United States), according to the manufacturer’s instructions. In consideration of the requirements of downstream processing, the gDNA was normalized to 1 ng/μL and stored at −20°C until amplification. DNA libraries were constructed using an Eltbio^®^ WHO_MT Kit (LD640), according to the manufacturer’s instructions. PCR amplification was performed in a final volume of 30 μL containing 10 ng of gDNA, 5 μL of RealCapChrMT Mix, and 10 μL of 3 ×EnzymeHF. Total reaction volumes were adjusted with nuclease-free water. The PCR conditions were as follows: enzyme activation for 3 min at 98°C, 13 cycles of 20 s at 98°C and 4 min at 58°C, seven cycles of 20 s at 98°C and 1 min at 72°C, and extension for 2 min at 72°C, followed by a 10°C hold. After purification, a second round of PCR amplification was performed to introduce adapters and barcodes. The reaction volume (30 μL) comprised 18 μL of purified products from the first round, 10 μL of 3 ×EnzymeHF, 1 μL of primer mix, and 1 μL of barcode mix. The PCR conditions were as follows: enzyme activation for 2 min at 98°C, six cycles of 15 s at 98°C, 15 s at 58°C and 15 s min at 72°C, and extension for 2 min at 72°C, followed by a 10°C hold. After purification, the libraries were pooled to a final concentration of 20 pM. Sequencing was performed on the Illumina HiSeq X Ten platform (Illumina, San Diego, CA, United States) with the corresponding Reagent Kit (PE150).

### 2.3 Statistical analysis

The sequence data obtained from the Illumina HiSeq X Ten platform (Illumina, San Diego, CA, United States) were automatically analyzed by base recognition and converted into the original sequences in the FASTQ format. First, we removed redundant primers and indexes from the initial offline data using Cutadapt v3.2 software (Martin, 2011). Second, low-quality reads were filtered using Trimmomatic v0.39 software (Bolger et al., 2014). To ensure successful alignment of the loop amplification captured sequence, the final cleaned files were mapped to the revised Cambridge Reference Sequence (Andrews et al., 1999) plus 64 bp (rCRS + 64 bp) using the Burrows–Wheeler Aligner (Li and Durbin, 2010) to generate the binary alignment/map (BAM) file. The sequences were also compared with the human reference genome hg19 to filter nuclear copies of mtDNA (NUMTs) (Just et al., 2015). bedtools v2.28.0 (Quinlan and Hall, 2010) was used to extract all reads that were successfully mapped to the hg19 reference genome from the BAM files in the previous step and then realigned to rCRS + 64 bp to generate new BAM files using Bowtie2 v2.3.5 software (Langmead and Salzberg, 2012). Then, we used Samtools v1.8 (Li et al., 2009) and VarScan v2.4.0 (Koboldt et al., 2009) to identify the mutation sites and output variants in VCF format files. Finally, BCFtools v1.9 (Li et al., 2009) was used to generate the consensus sequence (FASTA). FastQC was used to perform quality checks on each sample, and then MultiQC v1.9 software was used to integrate the quality inspection results of all samples.

### 2.4 Haplogroup assignment, genetic diversity analysis, and population comparisons

The haplogroup assignment of the whole mitochondrial genome generated from this study was carried out using HaploGrep 2 (Weissensteiner et al., 2016) based on PhyloTree build 17 (http://www.phylotree.org/tree/index.htm) and reconfirmed using the updated query engine (SAM2) built into EMPOP (Huber et al., 2018). The variants and haplogroup information of this study are listed in Supplementary Table S1. The haplogroup frequencies were calculated by using the direct counting method. Molecular diversity indices, haplotype diversity (HD), Tajima’s D, and Fu’s Fs were calculated using Arlequin version 3.5 software (Excoffier and Lischer, 2010). In order to detect population fluctuation, we obtained the Bayesian skyline plots (BSPs) based on complete mtDNA sequences using BEAST v2.0 software (Bouckaert et al., 2019) with a strict molecular clock. The analysis was performed with the mutation rate of 2.5 × 10^−8^ substitutions per nucleotide per year for the entire mitogenome, which was estimated based on 45,000-year-old Ust-Ishim (Wong et al., 2017). Tracer v1.7.2 (Rambaut et al., 2018) was used to analyze the data generated by BEAST.

To further investigate the genetic structure and affinity of Lhasa Tibetans, we collected the maternal genetic information of 107 East Asian populations from previous studies (Supplementary Table S2). The haplogroup status was re-determined using HaploGrep 2 with PhyloTree build 17 and reconfirmed using EMPOP. Principal component analysis (PCA) was carried out using MVSP version 3.21 software based on haplogroup frequencies. The pairwise Fst values between the Lhasa Tibetans and 107 reference populations were determined using Arlequin version 3.5 (Excoffier and Lischer, 2010), and a neighbor-joining (N-J) tree was further generated in MEGA X software (Kumar et al., 2018) based on the Fst values. The cmdscale function in R was used to generate multidimensional scaling (MDS) plots of the 108 Eurasian populations based on genetic distance matrixes. The visualization of all results was carried out based on the R platform, and the R packages “ggplot2” and “ggtree” were used for PCA, MDS, N-J tree, and Sankey plot visualization.

## 3 Results

The average number of mapped reads was 673,968.04 per sample, and the overall mean read depth was 4,862.68X ± 3,561.81X (mean ± SD) per individual. The scores of the mean quality and per sequence quality were over 30, implying that the quality of the sequencing data was good (Supplementary Figure S1). The value of HD was 0.9977 ± 0.015. The number of segregating sites (432), mean pairwise difference (MPD, 28.2761 ± 12.4432), and nucleotide diversity value (0.0017 ± 0.0008) were informative regarding the effective population size of Lhasa Tibetans. The values of Tajima’s D (−2.1082) and Fu’s Fs (−23.8051) neutrality tests showed significant negative results in Lhasa Tibetans, indicating possible recent population expansions or positive selection.

**FIGURE 1:**
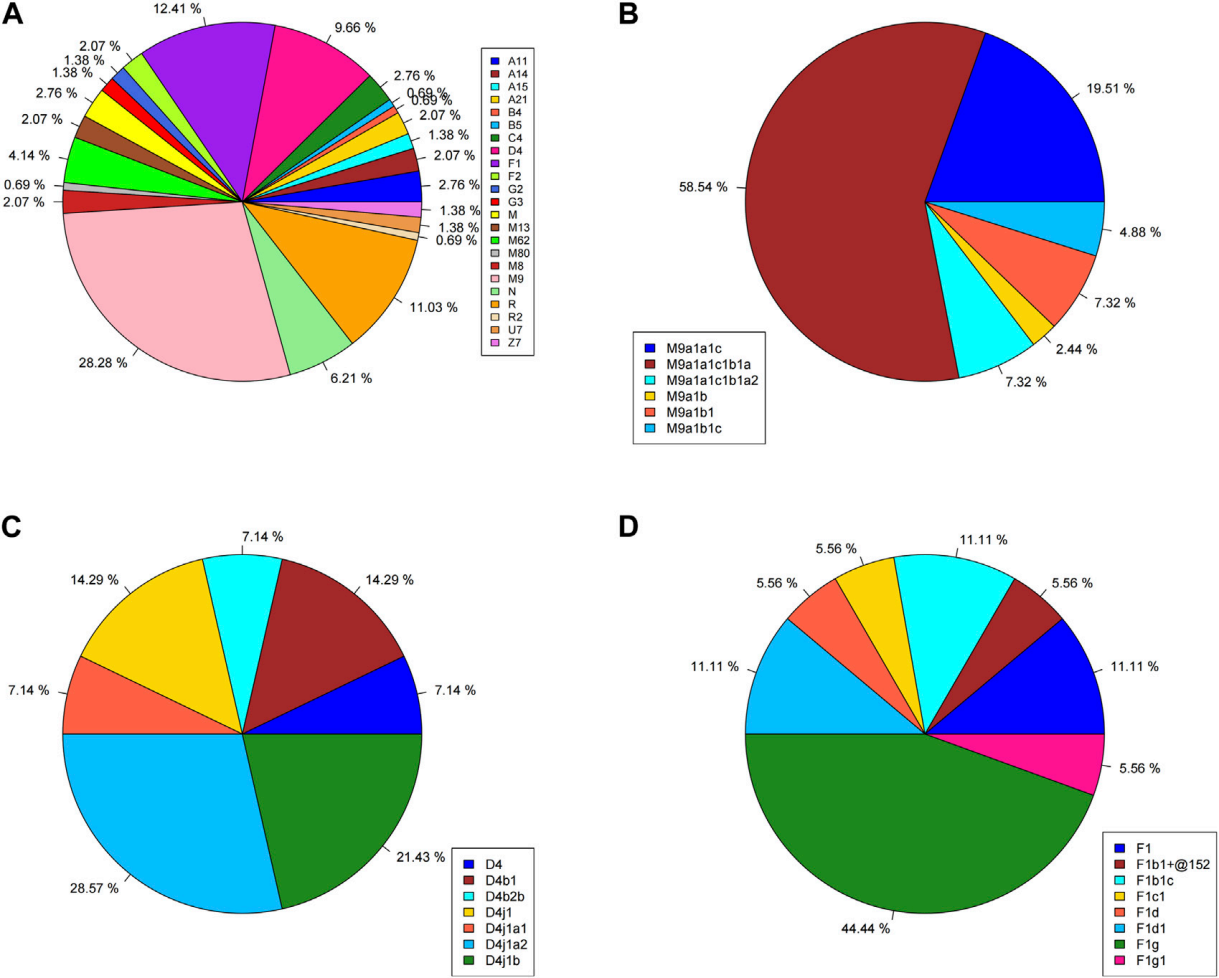
Haplogroup profiles of 145 Lhasa Tibetans. **(A)** Whole haplogroup distribution; **(B)** distribution of haplogroup M9; **(C)** distribution of haplogroup D4; **(D)** distribution of haplogroup F1.

The mtDNA haplogroup information of 145 Lhasa Tibetans is shown in Table 1 and Figure 1. The majority of the matrilineal haplogroups belonged to eastern Eurasian groups, and M9 was the most frequent haplogroup (28.28%), followed by R (11.03%), F1 (12.41%), and D4 (9.66%). Some maternal lineages were sporadically distributed among Lhasa Tibetans, such as haplogroups B4, B5, U7, Z7, G2, and G3. We found that M9a1a1c1b1a (58.54%) had the largest proportion in the M9 haplogroup, and F1g (44.44%) and D4j1a2 (28.57%) were the most frequent sub-haplogroups in F1 and D4, respectively. In this study, we explored the distribution of these haplogroups among East Asian populations (Supplementary Table S3). Haplogroup M9 showed higher frequency in highland Tibeto-Burman speakers, such as Tibetans, Lhoba, Deng and Sherpa, relative to other East Asian populations, and it also appeared in northern Han people and Tu, Yugur, Salar, and Bonan in Gansu. Haplogroups F1 and D4 appeared widely in East Asia, but F1 showed higher frequency in southern East Asian populations and Tibetans, and D4 was common in northern East Asian populations and distributed irregularly in Tibetans. Haplogroups N, M, R, B4, and B5 were common in southern lowland populations and showed lower frequency in Lhasa Tibetans. Haplogroups C4, G2, G3, and Z exhibited higher frequency in Tibetans and northern East Asian populations, such as Han, Mongolic, Tungusic, and Turkic speakers. Haplogroups A11, A14, A15, A21, and M80′D were shared by highland Tibeto-Burman speakers and Han people. Haplogroups U7 and R2 were shared by Tibetans and northern Han people and Turkic speakers. Haplogroups M62 and M13 were specific in highland Tibeto-Burman speakers. Haplogroups F2 and M8 exhibited low frequency in East Asian populations including Lhasa Tibetans.

**TABLE 1:** mtDNA haplogroups of 145 Lhasa Tibetans.

Haplogroup	Number of haplogroups	Frequency
A11	4	2.76
A14	3	2.07
A15	2	1.38
A21	3	2.07
B4	1	0.69
B5	1	0.69
C4	4	2.76
D4	14	9.66
F1	18	12.41
F2	3	2.07
G2	2	1.38
G3	2	1.38
M	4	2.76
M13	3	2.07
M62	6	4.14
M80′D	1	0.69
M8	3	2.07
M9	41	28.28
N	9	6.21
R	16	11.03
R2	1	0.69
U7	2	1.38
Z7	2	1.38

**FIGURE 2:**
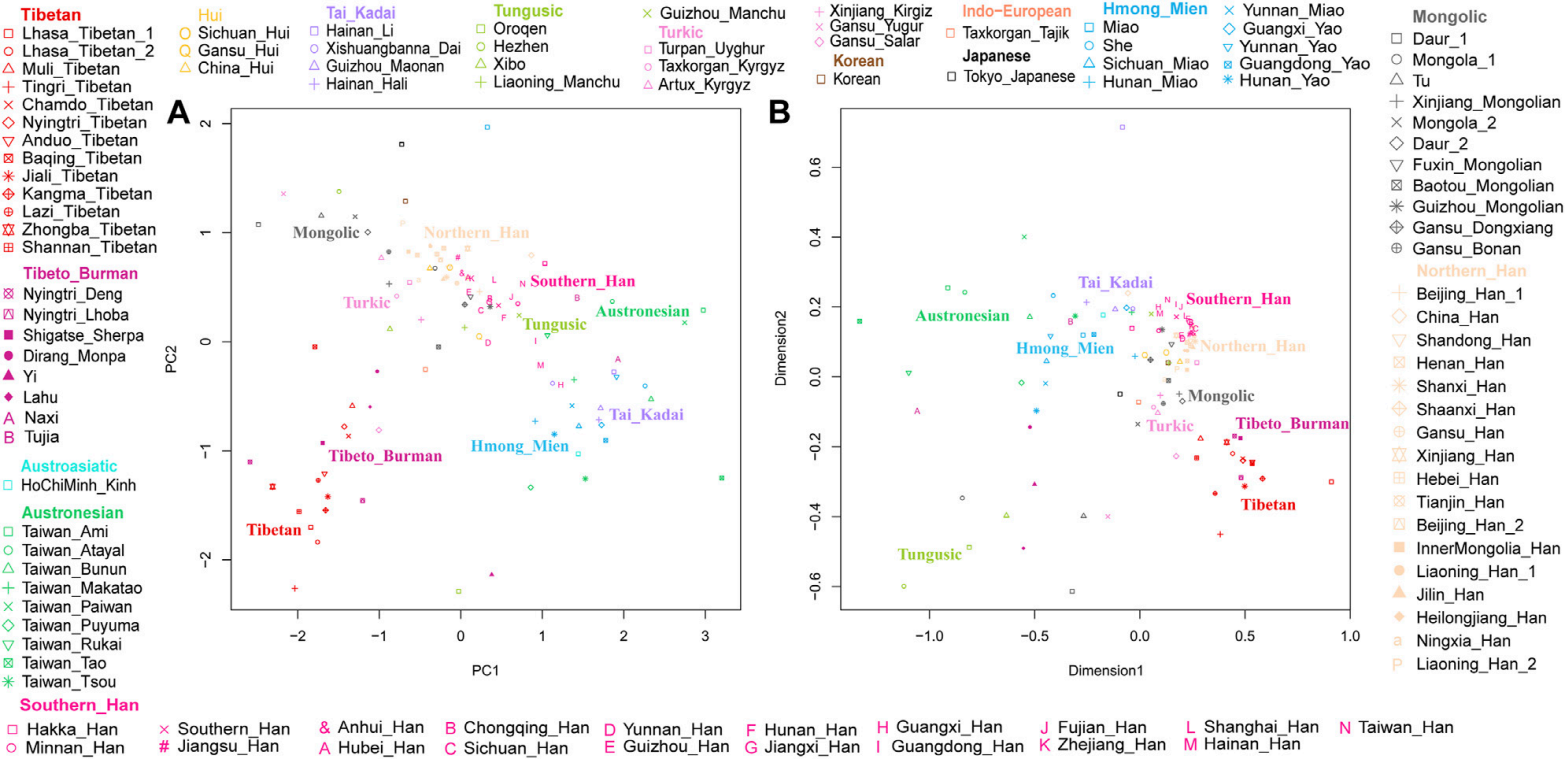
Genetic structure among Lhasa Tibetans and 107 East Asian populations. Populations are color-coded on the basis of their language family categories. **(A)** Principal component analysis; **(B)** multidimensional scaling plot.

The results of PCA and MDS are shown in Figure 2, and different colors represent different language groups. We found that population structure was related to the linguistic category and geographic distribution, there is an obvious north-to-south population stratification of East Asian populations, and same ethnolinguistically population clustered together. As shown in Figure 2A, Han people formed a north-south cline, while in Figure 2B, they clustered together, making it difficult to clearly identify the difference between northern and southern Han people. The southern Han population showed small genetic differences with Tai–Kadai-, Austronesian-, and Hmong–Mien-speaking populations, and Mongolic, Tungusic, and Turkic speakers clustered together and displayed short distances from northern Han people. Lhasa Tibetans and other highland Tibeto-Burman-speaking populations clustered together and far from other populations, and lowland Tibeto-Burman speakers distributed dispersedly and were adjoined to southern East Asian populations, but Tibetans showed genetic similarity with northern East Asian populations, indicating the north–south structure of Tibeto-Burman-speaking populations.

**FIGURE 3:**
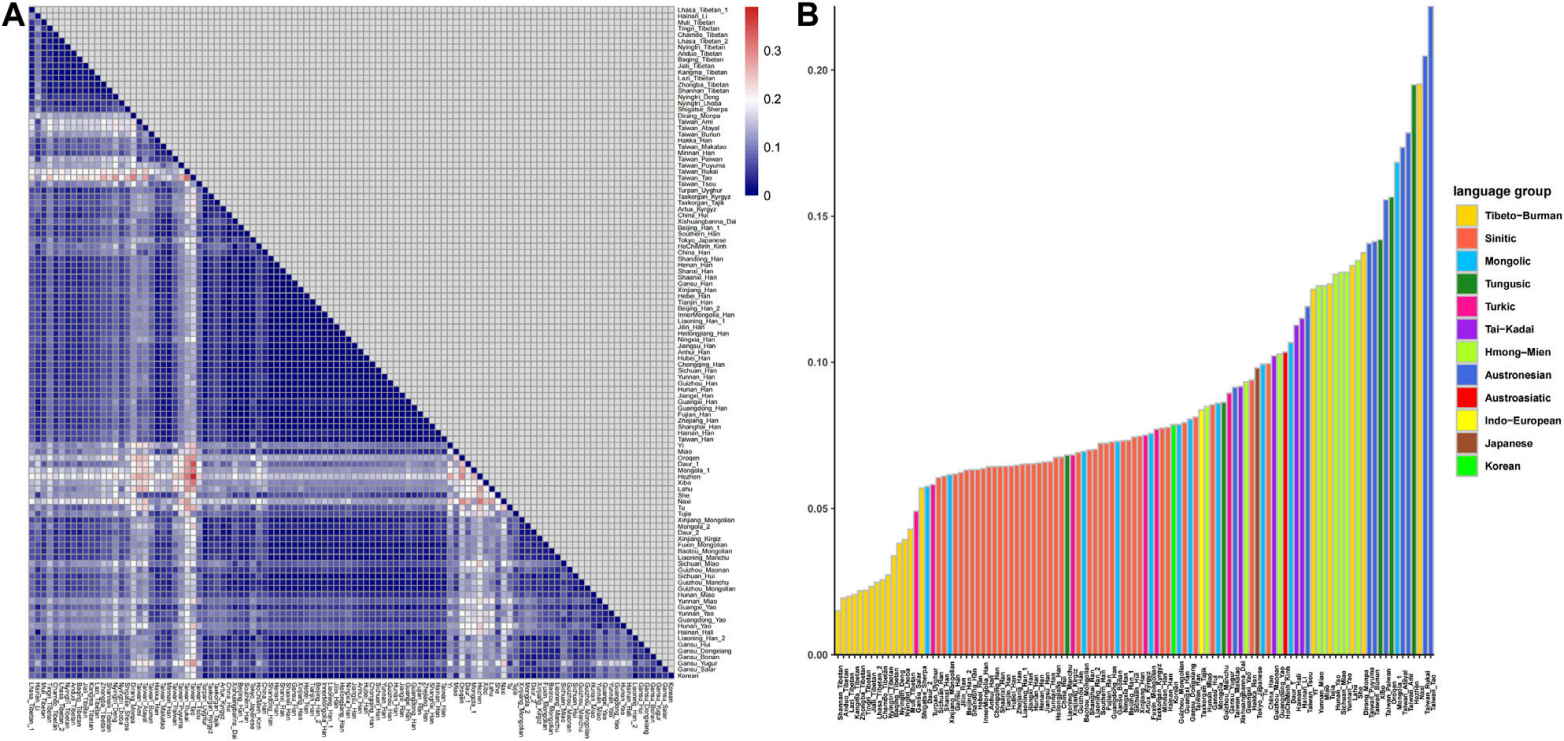
Pairwise Fst genetic distances between Lhasa Tibetans and 107 East Asian populations. **(A)** Heatmap based on pairwise Fst values; **(B)** bar chart of Fst values.

The results of pairwise Fst genetic distance among East Asian populations are shown in Figure 3 (Supplementary Table S4). As shown in Figure 3A, the deep blue color indicates short genetic distances between two populations. We found that the results were consistent with those of PCA and MDS, and populations from the same language group and adjacent geographic locations displayed short genetic distances. The Fst values between Lhasa Tibetans and other populations are shown in Figure 3B. Lhasa Tibetans had shorter genetic distances with other highland Tibeto-Burman speakers relative to other East Asians. Genetic homogeneity and shorter genetic distances were observed among Tibetans in Xizang relative to Sherpa (0.05697), Deng (0.0381), and Loba (0.03942), and the Lhasa Tibetans in this study showed the greatest genetic distances from Muli Tibetans (0.04291) among all referenced Tibetans. Linguists divided the Tibetan language into three sub-branches, Ü-Tsang, Ando, and Kham; however, if we subdivide this language, there are more than a dozen branches, and due to complex population migration and admixture, many Tibetans speak language other than Tibetan, such as Muli Tibetans in Sichuan, who speak the Pumi language. A previous study found that the sub-structure of Tibetans is related to the cultural background and geographic terrain (He et al., 2021), so the genetic difference between Lhasa Tibetans and Muli Tibetans can be attributed to different cultural backgrounds and geographic terrains and different population histories. We also observed short genetic distances between Lhasa Tibetans and populations with adjacent geographic locations, such as Salars in Gansu and Mongolians and Uyghurs in Xinjiang, and the obvious genetic similarity was observed between the Tibetan and Han people, especially the northern Han people (Shanxi, Gansu, Jilin, and Beijing).

**FIGURE 4:**
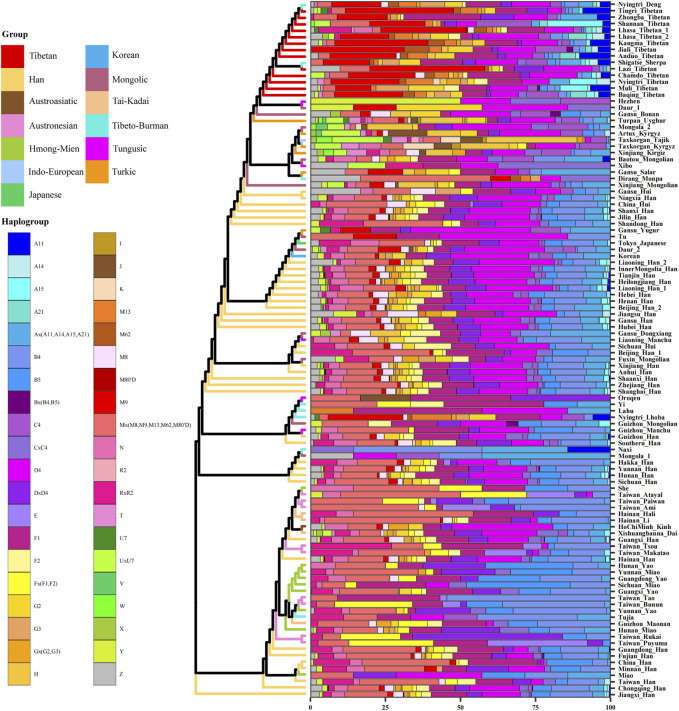
Maternal genetic structure and makeup of 108 East Asian populations.

Additionally, we constructed a phylogenetic tree of East Asian populations and showed the maternal genetic makeup of different people in Figure 4. The different linguistic groups and genetic components were marked by using different colors. The results were similar with PCA and MDS, and the population structure was related to the linguistic category. Han people in East Asia were divided into two groups: northern Han people clustered with Mongolic, Turkic, and Tungusic speakers, and southern Han people were close to Tai–Kadai-, Austronesian-, Hmong–Mien-, and Austroasiatic-speaking populations. All Tibetans formed a branch and clustered with other highland Tibeto-Burman speakers. Tibetans showed obvious northern East Asian characteristics, and northern Han people and Mongolic, Turkic, and Tungusic speakers in Northwest China were adjacent to Tibetans, further supporting the northern origin of Tibetans (Zhang et al., 2019; Wang C. C. et al., 2021). We also found some specific maternal haplogroups in Tibetans, such as A11, A21, M9, M13, and M62. The higher frequencies of these haplogroups in Tibetans are related to high-altitude adaptation (Gu et al., 2012; Kang et al., 2016).

To estimate the population size fluctuation over time, BSPs were performed for Lhasa Tibetans (Supplementary Figure S2). Previous studies have indicated that the initial colonization of the TP was in the early Upper Paleolithic (Qi et al., 2013; Lu et al., 2016). In BSPs, before 20,000 years, the population size was small and stable due to a limited productivity and unsuitable environment. The sharp expansion has occurred after the Last Glacial Maximum. During the Neolithic period, there is a continuous growth of an effective population size with agricultural development. After 5,000 years, there is an obvious expansion, which may be attributed to the introduction of barley agriculture and migration of Han people and their ancestors into the TP (Qi et al., 2013; Chen et al., 2015).

## 4 Discussion

Two root maternal haplogroups (M and N) were distributed mainly in East Asians. In this study, haplogroup M was found in 56.55% and haplogroup N in 43.44% of Lhasa Tibetans, and the maternal genetic landscape of Tibetans showed obvious East Asian characteristics, where M9a (28.28%), R (11.03%), F1 (12.41%), D4 (9.66%), N (6.21%), and M62 (4.14%) were the dominant haplogroups. M9a is the most prevalent maternal haplogroup in Tibetans, with frequencies ranging from 12.26% to 29.24% (Qi et al., 2013). Our results supported this conclusion. The M9a lineage can be further divided into two sub-haplogroups, M9a1a (85.37%) and M9a1b (14.63%), and M9a1a1c1b1a is the most frequent maternal haplogroup in Lhasa Tibetans. Ding et al. (2020) proposed that haplogroup M9a1a1c1b1a spread from Nepal to Tibet between 2,125 and 1,100 years ago, and Peng et al. (2011) reported that the M9 haplogroup might have originated in southern China and/or Southeast Asia and was related to migration around the Last Glacial Maximum in East Asia. Paleogenomic studies indicated that the earliest origins of haplogroup M9 can be dated back to the 14.0 kya Red Deer Cave individual located in Southwest China (Zhang X. et al., 2022), and haplogroup M9a can be found in Qingtai people related to the Yangshao culture (∼5,500–5,000 BP) (Miao et al., 2021). M9a1a1c1b1a in modern Tibetans is a genetic legacy of Neolithic millet farmers (Li, 2019), and haplogroups M9a1a and M9a1b showed that ancient Tibetans shared a common ancestor with ancient Middle and Upper Yellow River populations around the Early and Middle Holocene (Zhang et al., 2023).

Haplogroup D4 is prevalent in East Asia, especially in northern East Asian populations, and has the highest frequency in the modern Han population. It may have originated in northern China and may be associated with millet famers in the YRB, and the distribution of haplogroup D4 in East Asia could be attributed to the expansion of ancient agriculture (Li et al., 2019). Haplogroup D4 in Lhasa Tibetans could be further subdivided into D4j1 and D4b, and these two haplogroups also appeared in ancient Tibetans. D4j1 is shared between ancient Tibetans and Neolithic Zongri individuals (4,800–4,600 BP), and D4b appears in Neolithic Shimao and Miaozigou individuals. These haplogroups suggest a connection between highland ancient Tibetans and populations in the upper Yellow River region (Ning et al., 2020; Xue et al., 2022; Zhang et al., 2023). D4j1a2 and D4j1b are common haplogroups in Lhasa Tibetans, and D4j1b can be found in ancient Zongri individuals (Wang et al., 2023), revealing population movement from lower elevations to the TP between 4,750 and 2,775 years ago (Ding et al., 2020).

Haplogroup F1 is frequent in southern and southwestern China (Li et al., 2019). F1g is prevalent in Lhasa Tibetans, and it can be found in ancient Tibetans from 3.0 to 1.1 kya (Zhang et al., 2023) and ancient individuals related to the Qijia culture (3,800–4,000 BP) (Ning et al., 2020) and from the Wuzhuangguoliang site (5,400–4,800 BP) (Wang C. C. et al., 2021). F1g generated in lowlands is often found in Tibetans, revealing that the lowland population migrated to the TP (Kang et al., 2016). There were also some specific maternal lineages of Tibetans, such as A11a, A21, M13a, and M62, but these lineages were not prevalent in Lhasa Tibetans. Haplogroups A11a and A21 were found in ancient Tibetans during 3.0–1.1 kya (Zhang et al., 2023), and A11a1a in modern Tibetans was a genetic legacy of Neolithic millet farmers (Li, 2019). Haplogroups M13a and M62 migrated into the TP region with Late Pleistocene settlers during 40–20 kya (Zhao et al., 2009; Qin et al., 2010; Zhang et al., 2023).

**FIGURE 5:**
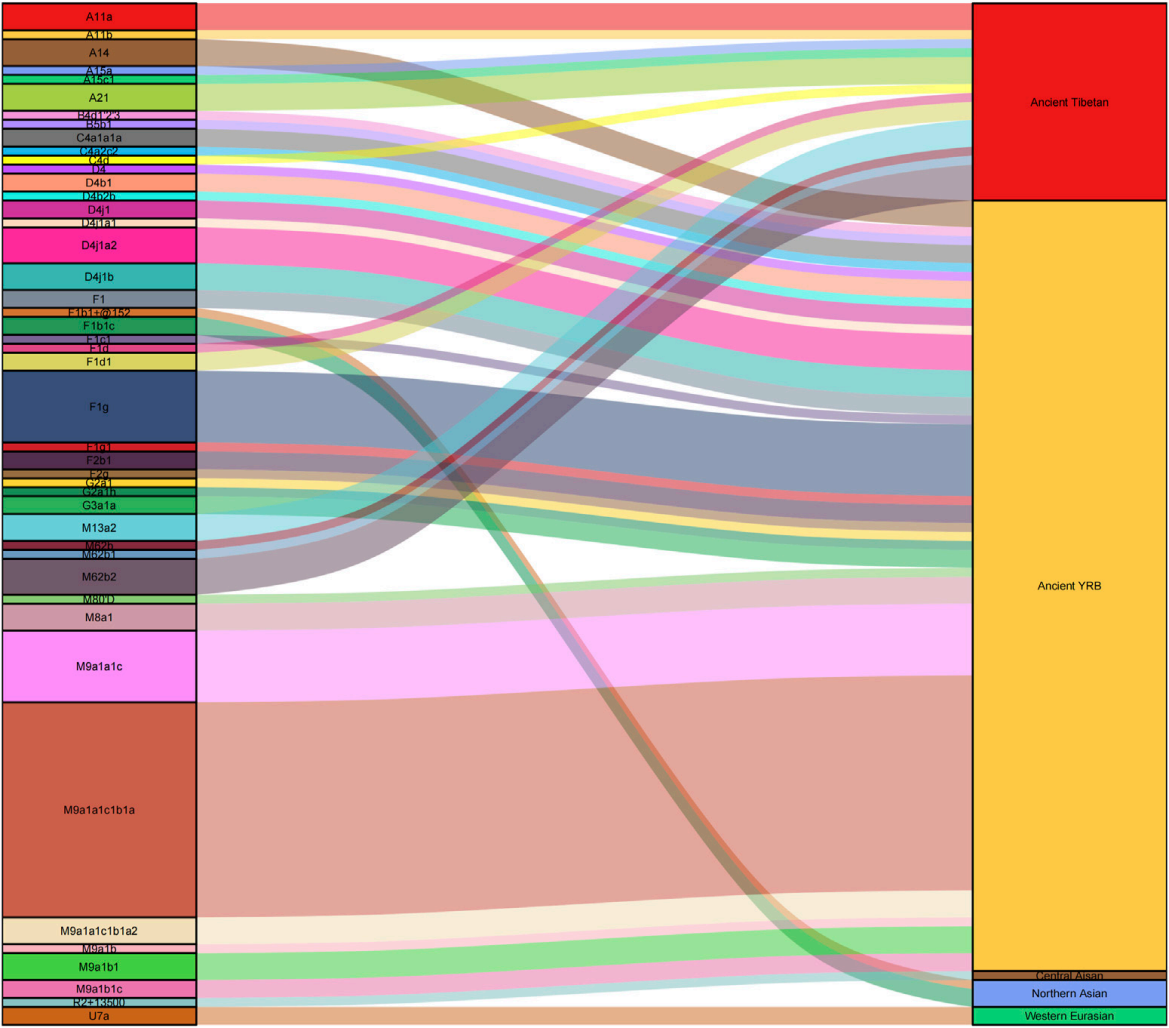
Potential sources of maternal genetic components in Lhasa Tibetans.

In this study, we found that some Tibetans belong to haplogroups N and R, which are the main macrohaplogroups in Eurasia. Studies of ancient DNA have found haplogroups N and R in ancient populations related to the Lower Xijiadian culture (∼4,000 BP), Majiayao culture (∼3,900 BP), Xiaohe culture (∼4,000–3,600 BP), Afanasievo culture (∼4,400 BP), Boisman culture (∼6,000 BP), and Ganj Dareh (∼9,900 BP) (Narasimhan et al., 2019; Ding et al., 2020; Ning et al., 2020; Wang C. C. et al., 2021; Zhang F. et al., 2021), indicating that Lhasa Tibetans have some ancient Eurasian ancestry and further supporting that the ancient Eurasian population contributed genetic components to modern Tibetans. There were also some maternal lineages sporadically distributed among Lhasa Tibetans, including haplogroups A15 (1.38%), A14 (2.07%), B4d1′2′3 (0.69%), B5b1 (0.69%), C4 (2.76%), F2 (2.07%), G2a1 (1.38%), G3a1 (1.38%), M80′D (0.69%), M8a1 (2.07%), U7a (1.38%), and Z7 (1.38%). Paleogenomic research revealed the possible sources of these maternal lineages. Haplogroups B5b1, G2a1, and M80′D existed in ancient people related to the Neolithic Shimao culture (3,977–3,699 BP) (Ning et al., 2020; Xue et al., 2022), and G2a1 could also be found in Neolithic Zongri individuals (Wang et al., 2023). Haplogroup C4a1a1 appeared in Neolithic Yangshao individuals (Miao et al., 2021). Haplogroup B5b1 was related to the Lower Xiajiadian culture during the Bronze Age (3,614–3,494 BP) (Ning et al., 2020). Haplogroup F2g was associated with the Neolithic Majiayao culture (3,800 BP) (Ding et al., 2020). Haplogroup A14 was related to the Neolithic Miaozigou culture (5,500 BP) (Ning et al., 2020). Haplogroup M8a could be found in ancient populations related to the Shimao culture, Taosi culture (∼4,150–3,696 BP), and Yangshao culture (Ning et al., 2020; Xue et al., 2022). Haplogroup B4d1′2′3 appeared at the LuoHeGuXiang site (2,338–2,180 BP) (Ning et al., 2020), and it also existed in ancient Tibetans during the same period (Wang et al., 2023). Haplogroup G3a1 could be found in ancient Xinhua individuals (4,231–3,650 BP) (Ding et al., 2020), and haplogroup G3a2 existed in Neolithic Shimao and Qijia individuals (Ding et al., 2020; Ning et al., 2020). In addition, G3a1 also appeared in ancient Dulan individuals during the Tubo Empire (Zhu et al., 2022). Haplogroups A15a and A15c1 existed on the TP 2,500–2,800 years ago (Ding et al., 2020). Previous studies based on ancient and present-day genomes supported the North-China-origin hypothesis of the Sino-Tibetan language family and dispersal patterns of northern millet farmers (He et al., 2021; Zhu et al., 2022). In this study, we found that the ancient populations related to the Yangshao, Qijia, Majiayao, Shimao, Miaozigou, and Zongri culture in the YRB provided substantial genetic contributions to modern Tibetans (Figure 5). Our results strongly support this hypothesis.

These sporadic lineages also revealed population interaction and admixture between Tibetans and adjacent populations. Haplogroups A, B, and C are some of the most common haplogroups in northern and eastern Asia (Derenko et al., 2007; Qin et al., 2010). Haplogroup A is rare although it has been found in many populations, with a frequency ranging from 5% to 10% (Derenko et al., 2007). Haplogroup A has the highest frequency in northern and northwestern China, and the frequency in Han people is 7% (Li et al., 2019). Previous studies have found that A15c1 is the specific maternal lineage of Sherpas (Wang et al., 2020). Haplogroup A15c1 could be found in Lhasa Tibetans, but only one individual belonged to this lineage, and the absence of A15c1 in modern Tibetans indicated the importance of the Tibetan-Yi Corridor in constructing the genetic diversity of Tibeto-Burman populations (Wang et al., 2020). Haplogroup B is divided into two main lineages: B4 and B5. Haplogroup B4 has a relatively high frequency in southern China (Li et al., 2019), B4 and B5 are also widespread in Southeast Asia and Taiwan (Trejaut et al., 2005; Duong et al., 2018), and B4 has a higher frequency among Austronesian speakers (Kim et al., 2012; Palencia-Madrid et al., 2019). Haplogroup C has a wide geographic distribution, and there are only two sub-lineages of C in Lhasa Tibetans (C4a and C4d). C4a can be found in different populations (Derenko et al., 2010; Duong et al., 2018), whereas the haplogroup C4d is considered a Tibetan-specific lineage (Qin et al., 2010); however, there is only one Lhasa Tibetan belonging to C4d in this study. Haplogroup Z is the characteristic of East Eurasian populations, and it appears in northern and southern China and is prevalent among Tibeto-Burman speakers (Zhao et al., 2011). In addition, this study also revealed an additional gene flow related to western Eurasian and Central Asian populations. Haplogroups U7a (1.38%) and R2 + 13500 (0.69%) could be found in Lhasa Tibetans. The geographical origin of haplogroup U7 is Europe, and this reveals matrilineal genetic continuity between Late Pleistocene hunter-gatherer groups and present-day populations of Europe and that haplogroup U7 spread to South Asia since the Holocene period (∼11.5 kya) (Sahakyan et al., 2017). It appeared among ancient Xinjiang people related to the Chemurchek culture during the Bronze Age (4,352–4,096 BP) (Wang W. et al., 2021; Kumar et al., 2022). Haplogroup R2 + 13500 appeared in ancient people in Central Asia and Xinjiang during the Iron Age (Narasimhan et al., 2019; Kumar et al., 2022). In general, our results indicated that Tibetans are an admixed population, and there are diverse genetic constituents related to East Asia, South Asia, North Asia, and western Eurasia in Lhasa Tibetans, which revealed a complex maternal genetic landscape. Due to different demographic histories, there are different patterns of adaption in highland people between the Pamir and TP (Peng et al., 2018), and more fine-scale genetic studies will play a pivotal role in understanding different population evolutionary histories.

In summary, this study supported the conclusion that the maternal genetic history of Tibetans can be characterized as long-term matrilineal continuity with frequent interactions between lowland and highland populations (Zhang et al., 2023). Prehistorical highland foragers, Yellow River millet farmers, ancient Tibetans, and present-day Han provided the majority of the genetic contributions to modern Tibetans. Multiple waves of lowland populations migrated to the TP during different periods, and the mitochondrial genome hinted at the roles of multi-ethnic group interaction and admixture events in shaping the genetic landscape of modern Tibetans.

## 5 Conclusion

This study investigated the whole mitochondrial genome of 145 Lhasa Tibetans and found that the maternal genetic landscape of Tibetans showed obvious East Asian characteristics, and M9a (28.28%), R (11.03%), F1 (12.41%), D4 (9.66%), N (6.21%), and M62 (4.14%) were the dominant haplogroups. This study further revealed long-time matrilineal continuity on the TP and a genetic connection between Tibetans and millet famers in the YRB and further supported the North China origin of the Sino-Tibetan language. We found that multiple waves of population interaction and admixture during different historical periods between lowland and highland populations shaped the maternal genetic profile of Tibetans.

## Data Availability

The original contributions presented in the study are publicly available. This data can be found in the China National GeneBank Database (https://db.cngb.org/), project accession number CNP0004942.
