# Supplementary figures and images for: Whole mitochondrial genome analysis in highland Tibetans: further matrilineal genetic structure exploration
Source: Front Genet. 2023 Nov 14;14:1221388. doi: 10.3389/fgene.2023.1221388 (PMC10682103; doi:10.3389/fgene.2023.1221388)

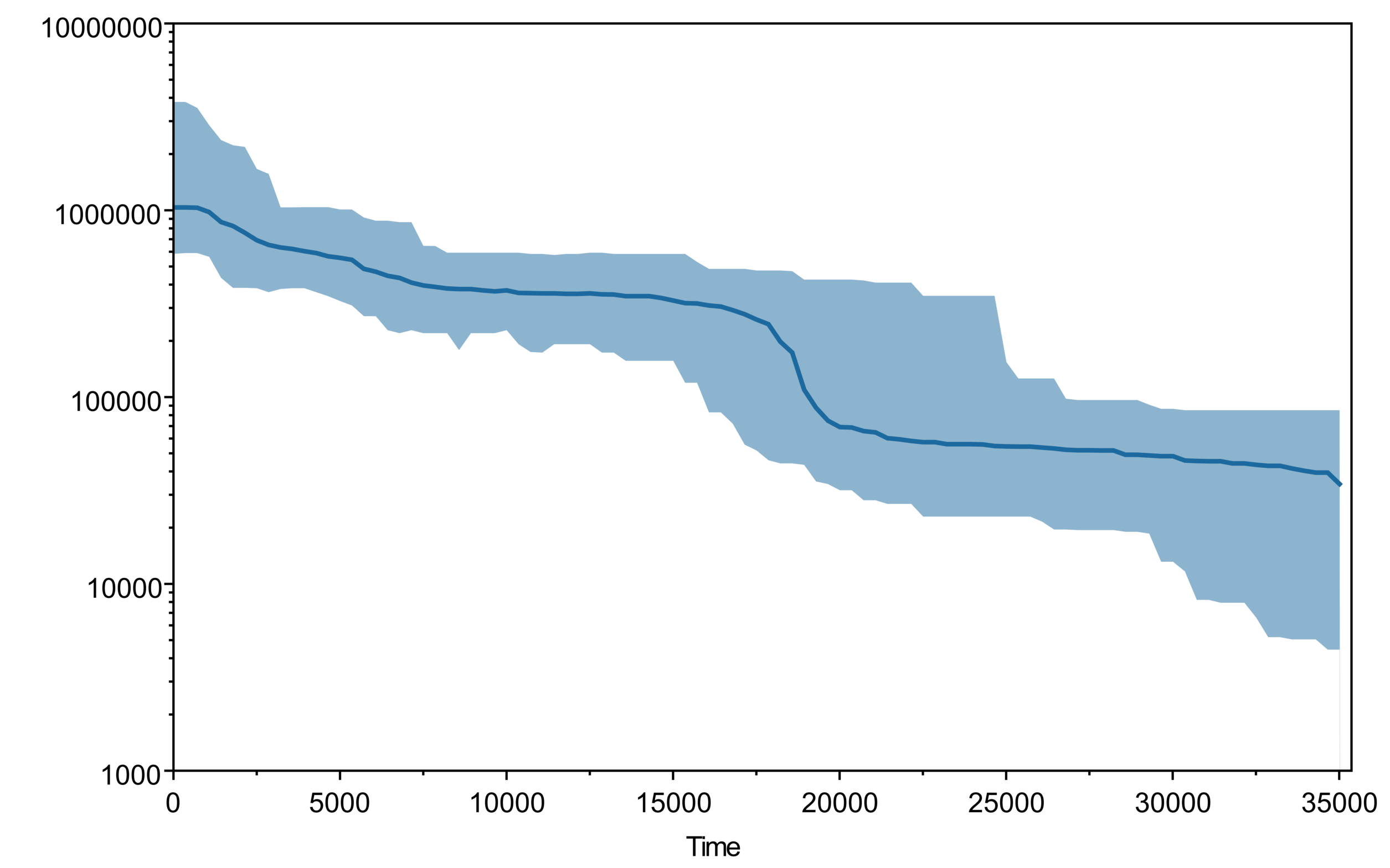

Supplement: Supplementary file 3 [file Image2.TIF]

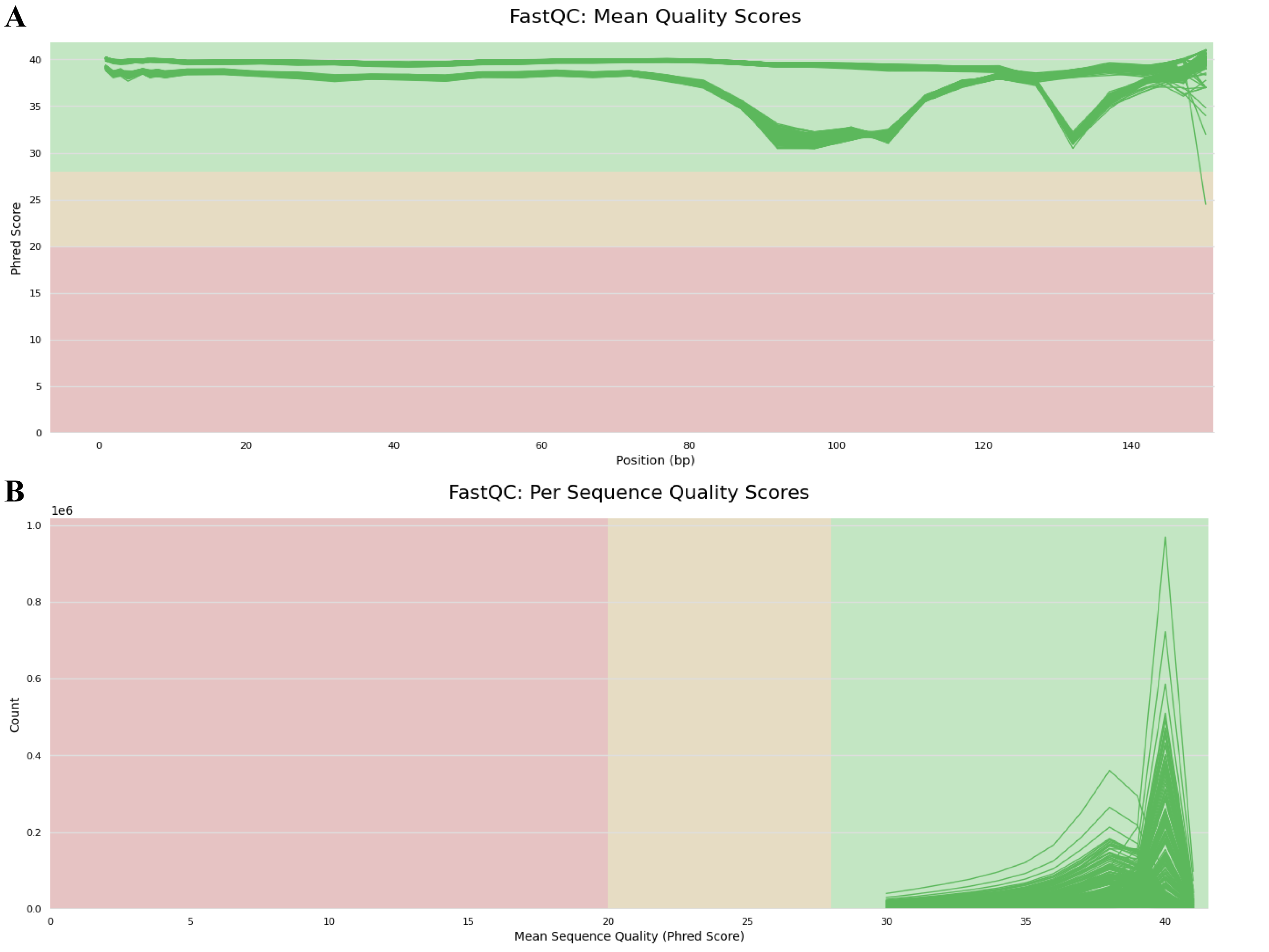

Supplement: Supplementary file 4 [file Image1.TIF]
